# An *in vivo* study of *Hypericum perforatum* in a niosomal topical drug delivery system

**DOI:** 10.1080/10717544.2018.1431977

**Published:** 2018-01-31

**Authors:** Mohamed Ali, Amira Abdel Motaal, Mohammed A. Ahmed, Abdulrhman Alsayari, Omaima N. El-Gazayerly

**Affiliations:** a Department of Research and Development, Atos Pharma, Cairo, Egypt;; b Department of Pharmacognosy, Faculty of Pharmacy, Cairo University, Cairo, Egypt;; c Department of Pharmacognosy, College of Pharmacy, King Khaled University, Abha, Kingdom of Saudi Arabia;; d Department of Pharmaceutics and Industrial Pharmacy, Faculty of Pharmacy, Cairo University, Cairo, Egypt

**Keywords:** *Hypericum perforatum*, niosomes, *in vivo*, standardization, wound

## Abstract

The active compounds present in *Hypericum perforatum* L. (Hypericaceae) include hyperforin, hypericins and flavonoids, which are assumed to be responsible for the activity of the extract in the treatment of wounds and scars. The present study aimed to incorporate *H. perforatum* extract standardized to a known content of phloroglucinols, naphthodianthrones and polyphenolic compounds into an effective transdermal drug delivery system capable of entrapping both lipophilic and hydrophilic constituents in the form of niosomal gels for wound treatment. An 80% ethanol extract (HE) was prepared on a pilot scale using DIG-MAZ. An HPLC-DAD holistic profile was established for HE and was standardized to contain 3.4 ± 4 rutin, 1.1 ± 3 chlorogenic acid, 0.5 ± 2 quercitrin, 2.8 ± 2 hyperforin, and 0.51 ± 3% w/w total hypericins. Niosomes were prepared using the modified reverse phase evaporation technique (REV). The wound healing effect of the gel was tested on 16 adult mongrel dogs. A significant decrease in the inflammatory cell count (18.4 ± 5.3) was recorded in the niosomal gel 1.5% NaCMC-treated group at the 7th day post wounding. It induced a marked regression in the inflammatory phase and enhanced the early beginning of the proliferative phase of wound healing. After 21 days, it showed complete re-epithelization, formation of new matrix fibers and significant reduction in the wound size, compared to the control and the Panthenol® 2% cream treated groups. This is the first study of *H. perforatum* in a niosomal topical drug delivery system.

## Introduction

*Hypericum perforatum* L. (Hypericaceae), commonly known as St. John’s Wort, is a herb which reaches a height of 50–60 cm. It is native to Europe, West Asia and North Africa and naturalized in many parts of the world, including North America, Australia and Chile. While it has been used as an antidepressant herbal drug, nevertheless, it has been traditionally known as an external remedy for wounds, ulcers, sunburns and hemorrhoids (WHO, 2002).

*Hypericum* contains flavonoids, hyperforins and the degradation product of hypericin, which provides its red color (Miraldi et al., 2006). The highly lipophilic hyperforin (phloroglucinols) possesses antimicrobial, anti-inflammatory, and antioxidant effects, as well as stimulating the differentiation of keratinocytes. The lipophilic hypericins (naphthodianthrones) also demonstrate antimicrobial and anti-inflammatory activities. Traditional preparations of *Hypericum*, which are composed of variable components, have strong dermatological effects which favor the synergistic activity of hyperforin, hypericins and the other hydrophilic components such as flavonoids and phenolic compounds (Wölfle et al., 2014).

Niosomes are novel drug delivery systems (Moghassemi & Hadjizadeh, 2014). They can entrap lipophilic drugs into vesicular bilayer membranes and capture hydrophilic drugs in aqueous compartments. They possess a higher penetrating capability than other preparations commonly used nowadays as transdermal drug delivery systems (Purasa et al., 2013).

Wound healing is the natural response of injured tissues, consisting of the interactive phases of inflammation, proliferation and remodeling. Several studies have investigated the wound healing activity of *H. perforatum* extracts and fractions; however, none of these reports established a holistic chromatographic fingerprint of the tested *Hypericum* extract showing its content of phloroglucinols, naphthodianthrones and phenolic compounds (Öztürk et al., 2007; Süntar et al., 2010). Süntar et al. (2010) proved that the aerial parts of *H. perforatum* possessed remarkable wound healing and anti-inflammatory activities by enhancing the migration of fibroblasts and collagen deposition, and that the ethyl acetate fraction containing flavonoids and naphthodianthrones possessed the highest activity. Öztürk et al. (2007) assumed that the wound-healing activity was due to fibroblast migration and the stimulation of collagen synthesis, speculating that it was not solely dependent on the hypericin content.

The pharmaceutical formulations available nowadays contain the extract, which is usually standardized to include only hyperforin, providing a lipophilic ointment in an oily base which is inconvenient and not very effective. Thus the aim of the present study was to prepare a niosomal topical gel formulation capable of entrapping both the lipophilic and hydrophilic constituents of *Hypericum* extract standardized to contain a specific concentration of hyperforin, hypercins and other phenolic compounds, and to study its *in vivo* wound healing effect on dogs.

## Materials and methods

### Plant material

The flowering tops of *H. perforatum* were collected in May 2010 from SEKEM farms, 3 Cairo-Belbeis Desert Road, Egypt. The plant was authenticated by Dr. M. Gebali (Plant Taxonomy and Egyptian Flora Department, National Research Center, Giza, Egypt). The flowering tops were dried in a vacuum oven at 40 °C. The plant name was checked using The Plant List (http://www.theplantlist.org/). A voucher specimen (voucher no. 916) was deposited at the herbarium of the Pharmacognosy Department, Faculty of Pharmacy, Cairo University, Egypt.

### General

Acetonitrile, methanol, ortho-phosphoric acid (HPLC grade), rutin, hyperoside, hypericin and hyperforin were purchased from Sigma-Aldrich, Germany for phytochemical profiling. Quercetin dihydrate (≥98%) was obtained from Carl Roth (Karlsruhe, Germany). Hydroxyethyl cellulose (HEC), hydroxy propylmethyl cellulose (HPMC), sodium carboxymethyl cellulose (NaCMC), and Pluronic F-127 were purchased from Sigma-Aldrich, Germany for the formulations. Ketamine HCl 5% (Rotex medica, Trittau, Germany), and Panthenol® 2% cream (Nile Co. for Pharmaceuticals and Chemical Industries, Egypt) were obtained for the *in vivo* experiments. Formaline, xylene, hematoxylin and eosin stain, and Masson’s trichrome stain were sourced from Sigma-Aldrich, Germany for the histopathology. All other chemicals and reagents were of analytical grade.

### Extraction using ASE®100

*Hypericum perforatum* dried flowering tops were extracted on a laboratory scale using accelerated solvent extraction ASE®100 (Dionex). The best extraction method was determined by choosing between methanol 80% and ethanol 80% as extraction solvents, both at room temperature and at 70 °C. The herb was cut with a mixer for a few seconds and the powder was dispensed into the extraction cell. The extracts were evaporated under a vacuum using the rotary evaporator at a temperature not exceeding 40˚C. The mean dry weight of the extracts was calculated as the average of three measurements ± S.E.

### Extraction using DIG-MAZ

The dried flowering tops were extracted on a pilot scale using a DIG–MAZ multifunctional extraction system (Samtech, Austria) which combines different methods of extraction such as percolation, digestion, maceration and distillation. The dried herb was milled to a particle size of 6 mm, a 10 L extraction vessel was packed with the herb (1.23 kg), and 80% ethanol was pumped into the 60 L solvent container at a constant temperature of 70 °C. A total of four extraction cycles were run using 15, 5, 5.1 and 4.2 L of fresh solvent for 90, 40, 40 and 45 min extraction times, respectively. The extract (HE) was concentrated under vacuum, then lyophilized (yield = 409 g) and stored at –20 °C, away from light and under nitrogen, for further use.

### Niosomes preparation

The niosomes were prepared using the modified reverse phase evaporation technique (REV) (Handa et al., 1987) with different ratios of Span 20, 60 and 80, where six niosomal systems were prepared (F1–F6) (Table 1(A)). The extract (HE) was dispersed in distilled water using a sonicator (Crest Ultrasonic Corp., Model 275 T, Trenton, NJ). Cholesterol and Span were dissolved in a mixture of chloroform and methanol (2:1) and stirred at 800 rpm using a mechanical mixer (Russell Hobbs, Austria). The prepared extract solution was added to the solvent and homogenized for 3 min. The suspension was then heated in a water bath for 10 min at 60 °C to evaporate the organic solvents.

**Table 1.** Niosomes: (A) composition of the niosomes; (B) encapsulation efficiency of niosomes (F1–F6). (A)

Sample	Span 20	Span 60	Span 80	Cholesterol	Extract
F1	1^a^	0	0	1	1
F2	0	1	0	1	1
F3	0	0	1	1	1
F4	0.5	0.5	0	1	1
F5	0.5	0	0.5	1	1
F6	0	0.5	0.5	1	1

a All values are given as weight ratio.

### Determination of niosome encapsulation efficiency

The niosomal suspension was centrifuged at 15,000 rpm at 4 °C for 20 min. The supernatant was removed and the precipitate was washed three times with a buffer to remove all the released compounds. The precipitate was then dissolved in 96% ethanol using sonication for 30 min (Ruckmani & Sankar, 2010). Quantitative analysis of the extract content of the niosomes was measured by HPLC-DAD and the total hypericins was determined spectrophotometrically. The encapsulation efficiency (EE%) was calculated by the ratio of the amount of entrapped compound in pellets to the initial amount in the used extract (HE).

### Particle size, zeta potential and morphology of optimal niosomal formula

The vesicle size and zeta potential of the optimal niosomal formula with the highest encapsulation efficiency was determined using Malvern Zetasizer at 25 °C (Malvern Instrument Ltd., Worcestershire, UK). The morphological aspects of the optimal niosomal formula was evaluated by transmission electron microscopy (TEM) (Jeol-200 CX, Joel).

### Preparation of niosomal gel

Gels were prepared by dissolving the gelling agent polymer in distilled water (total weight 50 g) and stirring at 270 rpm using a mechanical stirrer (Heidolph RZR 2021). Two grams of the optimal niosomal formula was dispersed in 38 g distilled water and added to the gel. Glycerin (2.5 g) and propylene glycol (5 g) were mixed and added to the gel as preservatives. Sodium carboxymethyl cellulose (NaCMC) 1.5% and hydroxyethyl cellulose (HEC) 3% were selected as polymers (results for selection are not shown).

### *In vitro* release of the niosomal gel

This study was carried out using a USP dissolution test apparatus 1 (Pharma test, Germany), with a slight modification in which the basket was removed and a stainless steel screen (mesh diameter =100 µm) was inserted instead. The specified weight of the niosomal gel to be tested was placed on the stainless steel screen and dipped in a 400 ml aqueous solution placed in the vessel of the USP dissolution test apparatus to maintain a sink condition. The release study was carried out at 32 ± 0.5 °C (skin temperature) and the stirring shaft was rotated at a speed of 50 rpm (Rafiee-Tehrani & Mehramizi, 2000). Aliquots of the released medium were withdrawn for analysis at predetermined time intervals (30, 60, 90, 120, 150 and 180 min), and replaced with equal amounts of fresh medium to maintain constant volume. The absorbance of the collected samples was measured spectrophotometrically at 590 nm, after filtration, using the aqueous solution as a blank. All experiments were done in triplicate.

The mechanism of release was determined. The release data were analyzed using a linear regression according to:Zero order kinetics: *C*_t_ = *C*_o_−*k*_o_*t*First order kinetics: *C*_t_ = *C*_o_*e*^−*kt*^

And according to the simplified Higuchi diffusion model:

$$C_{\text{t}}=k_{\text{h}}\surd t$$

where *C*_t_ is the total amount of the drug % released after time *t*, *C*_o_ is the initial amount of the drug %, *k*_o_ is the zero order release rate constant (% min^−1^), *k* is the first order release rate constant (min^−1^), and *k*_h_ is the rate constant obtained according to the Higuchi equation (% min^−0.5^).

The order of the drug release from each gel was determined by calculating the coefficient of determination (*r*^2^) in each case. The highest value represents the order of the drug release from the gel.

### HPLC analysis of extracts and gels

An analytical HPLC method was developed for the analysis of the extracts and niosomal gels. An UltiMate^®^ 3000 HPLC (Dionex, CA, USA) was used, equipped with a P680 LPG quaternary pump, and a UVD 340 U photodiode array detector operated using the CHROMELEON™ Chromatography Management System (Thermo Fischer Scientific, MA, USA). All samples were dissolved in methanol and filtered through a syringe filter (CHROMAFIL RC-45/25 0.45 µm, Carl Roth, Karlsruhe, Germany). The extracts and standards were injected into an Acclaim^®^ 120 C18, 5 µm, 150 × 4.6 mm column. The mobile phases used were 0.3% ortho-phosphoric acid in water (solvent A) and acetonitrile (solvent B). Gradient elution was carried out at a flow rate of 1.0 ml/min (0–12 min, 90:10 to 80:20 A:B; 12–30 min, 80:20 to 10:90 A:B; 30–37 min, 10:90 to 5:95 A:B; 37–40 min, 5:95 to 90:10 A:B; 40–45 min, 90:10 A:B). The injection volume was 20 µL and UV detection was performed at 270 nm. Quantitative analysis of the major compounds present in the extracts and niosomal gels was carried out utilizing relative response factors relative to rutin (Isacchi et al., 2007). A standard calibration curve of rutin was established (40–200 µg/mL). Standards and samples were injected in triplicates.

### Spectrophotometry

The total hypericins in the extracts and niosomal gels was determined using a Shimadzu UV-1601 spectrophotometer (EMEA, 2009). A solution of each sample was prepared in methanol at a concentration of 2 µg/mL. The test solution was filtered via syringe filter PTFE 0.45 µm (Sigma-Aldrich), and the absorbance was measured at *λ* = 590 nm against methanol as a blank. Total hypericins = A/(C × 870), where A is the absorbance at 590 nm, C is the concentration of the test solution in g/ml, and 870 is the specific absorbance of hypericin.

### Experimental animals

The experiments were carried out on 16 adult mongrel dogs of both sexes, weighing 20 to 25 kg. The animals were housed under standard environmental conditions (23 ± 1 °C, with 55 ± 5% humidity and a 12 h light/dark cycle), and maintained with free access to water and three meals per day, including dry food (Sportmix-adult^®^, IL, USA).

### *In vivo* experiment

**Figure 1.**
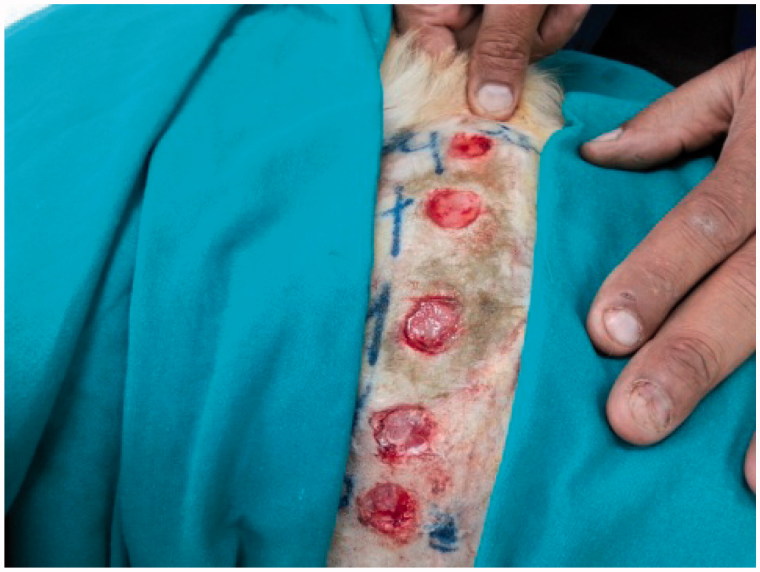
Skin wounds (2 cm in diameter) on one side of the thorax of the dog.

This study was carried out in accordance with the recommendations of the guidelines for the care and use of laboratory animals of the National Institutes of Health (USA). The protocol was approved by the Ethical Committee for Animal Use and Care, at the Faculty of Veterinary Medicine, Cairo University, Egypt. All surgeries were performed under general anesthesia, and all efforts were made to minimize suffering. The dogs were anesthetized by intravenous injection of 10 mg/kg Ketamine HCl 5% (Süntar et al., 2010). One side of the thorax of each dog was surgically prepared for aseptic surgery. Five full thickness circular skin wounds, 2 cm in diameter each, were performed (Figure 1). The animals were randomly divided into 4 main groups, each containing 4 dogs, representing 0, 7, 14 and 21 days after injury. The main groups were further subdivided into 4 subgroups according to the dressing materials, as follows: subgroup 1 – no material was used at the injured area as a control; subgroup 2 – each injured area was covered with 0.5 g Panthenol® cream three times daily; subgroups 3 and 4 – each injured area was covered with 0.5 g *Hypericum* niosomal gel 1.5% NaCMC three times daily. All clinical observations, measurements, and analysis of the results were taken by an investigator who was blind to the group allocation and experimental design.

### Wound area and % wound closure

The wound of each group was examined and the area of the wound was measured using a graduated ruler (Süntar et al., 2010) on the day of the surgery (0 day) and on the 3rd, 5th, 7th, 9th, and 14th days post-surgery. Then the percentage of wound closure was calculated, according to Ghaisas et al. (2014). Measurements were done three times and analyzed using SPSS V.17 software. Values were reported as mean ± standard error of the mean (SE).

% wound closure = (wound area at day 0-wound area at day n)/wound area at day 0 × 100

where *n*= number of days (0, 3, 5, 7, 9 and 14)

### Histolopathological study

Tissue specimens from the skin wound at 0 (2 hours post-surgery), 7th, 14th and 21st days post wounding were fixed in 10% formaline, dehydrated, cleared in xylene, then embedded in paraffin (Süntar et al., 2010). Tissue sections of 5 µm thickness were stained with hematoxylin and eosin stain (H&E) or Masson’s trichrome stain, and then examined under the light microscope (Leica Microsystems, Wetzler, Germany). H&E stained sections were examined to demonstrate inflammatory cell count, including polymorphnuclear and mononuclear cells, edema, hemorrhage, necrosis, fibroblast proliferation and angiogenesis. Masson’s trichrome-stained sections were examined to demonstrate granulation tissue and matrix formation.

### Morphometric analysis of inflammatory cells

Inflammatory cells (polymorphnuclear and mononuclear) were counted in five consecutive magnification fields (X40), from the superficial part of the wound gap on the 7th day post wounding (Süntar et al., 2010). The data was statistically analyzed using one-way analysis of variance (ANOVA), SPSS version 17 software. Values were reported as mean ± standard error of the mean (SE).

## Results

### Hypericum extract

**Figure 2.**
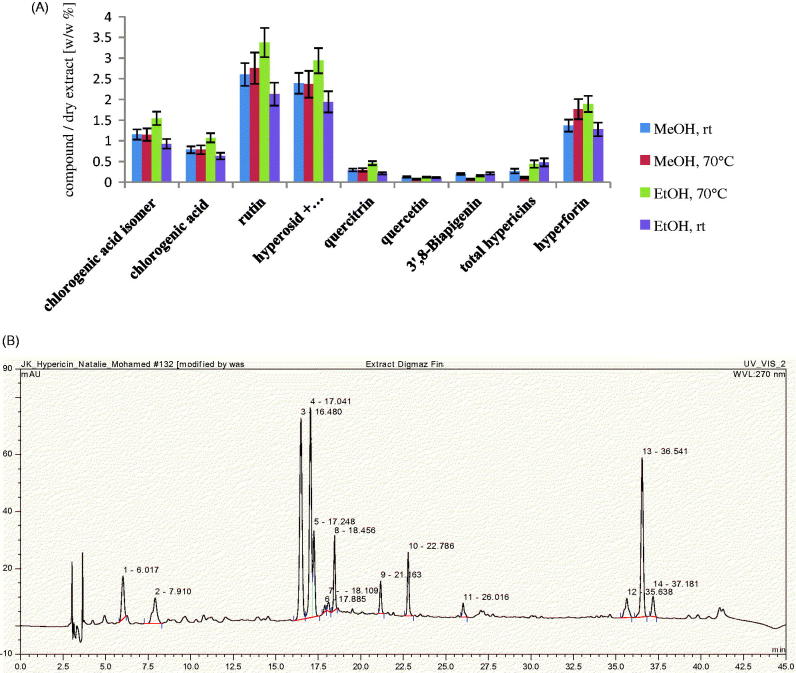
Phytochemical analysis. (A) Analysis of the extracts (ASE^®^100) using a Shimadzu spectrophotometer for total hypericins and HPLC-DAD for all other compounds. Hyperoside and isoquercitrin, being similar, were not separated under the present chromatographic conditions. MeOH: methanol; rt: room temperature; EtOH: ethanol. (B) HPLC-DAD chromatographic profile of the 80% ethanol extract at 70 ^°^C (HE) using DIG-MAZ. 1, chlorogenic acid isomer at Rt 6.02; 2, chlorogenic acid at Rt 7.91; 3, rutin at Rt 16.48; 4, hyperoside at Rt 17.04; 5, isoquercitrin at Rt 17.25; 8, quercitrin at Rt 18.46; 9, quercetin at Rt 21.16; 10, I3’, II8 biapigenin at Rt 22.79; 11, pseudohypericin at Rt 26.02; 12, hyperforin analog at Rt 35.64; 13, hyperforin at Rt 36.54; adhyperforin at Rt 37.18.

Alcohols are generally used for the extraction of a wide spectrum of polar and non-polar compounds. Methanol is the most commonly used solvent for the extraction and analysis of *H. perforatum* on a laboratory scale (Isacchi et al., 2007; Filippini et al., 2010; Tawaha et al., 2010). However, since this is classified as toxic, ethanol is preferable in pharmaceutical preparations. The extraction efficiencies of these two solvents were compared at room temperature and at 70 °C using the accelerated solvent extraction ASE^®^100; results revealed that the extraction yield of ethanol 80% at 70 °C was the highest (22 ± 3 w/w %) (Figure 1S). Also, comparative quantitative study of the major constituents in the four extracts showed that ethanol 80% at 70 °C yielded the highest amounts of active constituents, when viewed relatively (Figure 2(A)). Thus pilot scale extraction using DIG-MAZ was carried out using ethanol 80% (70 °C) as an extraction solvent. An HPLC-DAD chromatographic profile was established for the extract (HE) showing the major constituents (Figure 2(B)). HE was standardized to contain 3.4 ± 4 rutin, 1.1 ± 3 chlorogenic acid, 0.5 ± 2 quercitrin, 2.8 ± 2% hyperforin w/w and 0.51 ± 3% w/w of total hypericins, as determined spectrophotometerically at *λ* = 590 nm.

#### Analysis and drug entrapment of niosomes

HPLC and spectrophotometric analysis of the niosomes revealed that all prepared niosomes contained nearly the same concentrations of active ingredients as the incorporated extract (HE). The EE% for total hypericins, hyperforin, rutin and quercitrin were calculated for the 6 niosomes (Table 1(B)). The highest amount of hyperforin was encapsulated by the niosome F1, while that of total hypericins and quercitin by niosome F5 and rutin by niosome F6. However, the F1 niosome (Span 20:cholesterol, 1:1) showed relatively the highest content of total hypericins, hyperforin, rutin and quercitrin (0.42 ± 0.1, 2.1 ± 0.2, 1.2 ± 0.2 and 0.31 ± 0.02 w/w %, respectively). Also, 80% of the drug was entrapped in niosome F1, calculated as total hypericins. Thus F1 was used for preparation of the niosomal gel.

**Table:** (B)

Encapsulation efficiency [EE% ± S.E.]				
	Hyperforin	Hypericin	Rutin	Quercitrin
F1	70 ± 1.3	74 ± 1.4	28 ± 1.2	45 ± 0.7
F2	55 ± 2.0	35 ± 1.7	35 ± 1.5	75 ± 0.6
F3	61 ± 1.4	29 ± 0.9	13 ± 0.7	84 ± 0.9
F4	48 ± 1.4	88 ± 0.8	18 ± 0.9	92 ± 1.4
F5	40 ± 1.6	100 ± 2.1	14 ± 0.8	93 ± 1.7
F6	47 ± 1.3	90 ± 1.0	41 ± 1.3	90 ± 1.3

#### Particle size, zeta potential and morphology of optimal niosomal formula

Particle size measurement was done to confirm that particles of the optimal niosomal formula (F1) were all of nanometer range. The Z-average of F1 was found to be 490.45 ± 27.64 nm, with a polydispersity index of 0.26 ± 0.01. The measured zeta potential was found to be −41.10 ± 0.84. The obtained high zeta potential value indicated high stability of the prepared niosomal dispersion.

**Figure 3.**
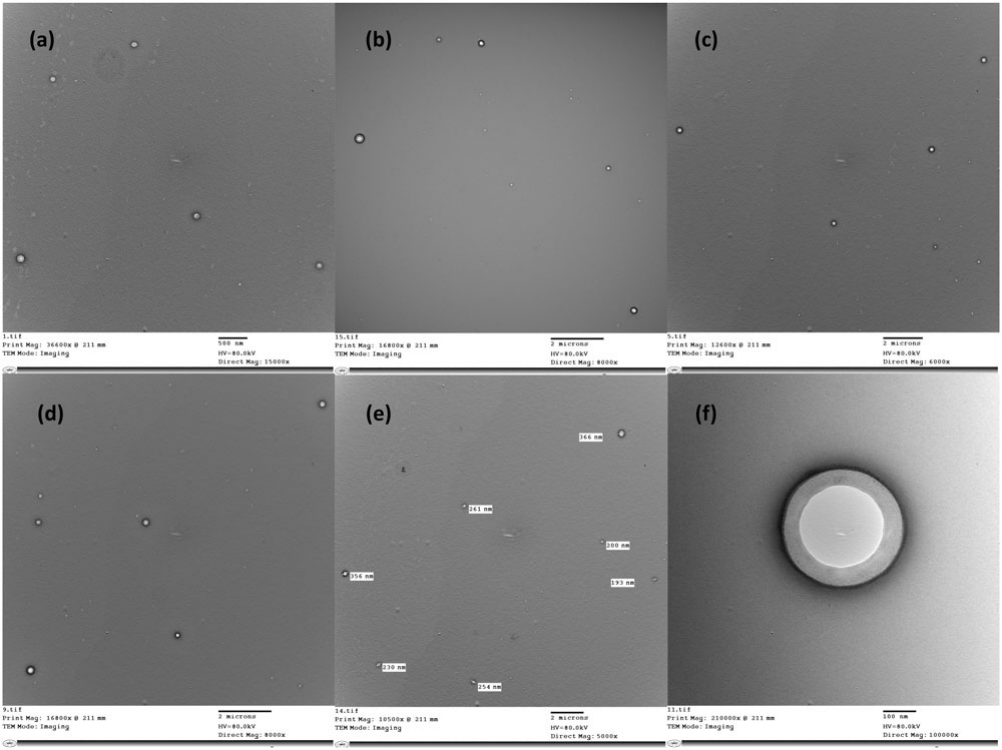
TEM micrographs of optimum niosomal formula (F1).

TEM micrographs of optimum niosomal formula (F1) are shown in Figure 3. The morphology was examined using TEM to confirm the formation of niosomal vesicles with a bilayer wall. The obtained TEM micrographs showed that the prepared niosomes were of nano-size, which confirmed the results of the particles size measurement. Micrographs also confirmed the formation of niosomal vesicles with bilayer membranes.

#### *In vitro* drug release of niosomes in different gel bases

**Figure 4.**
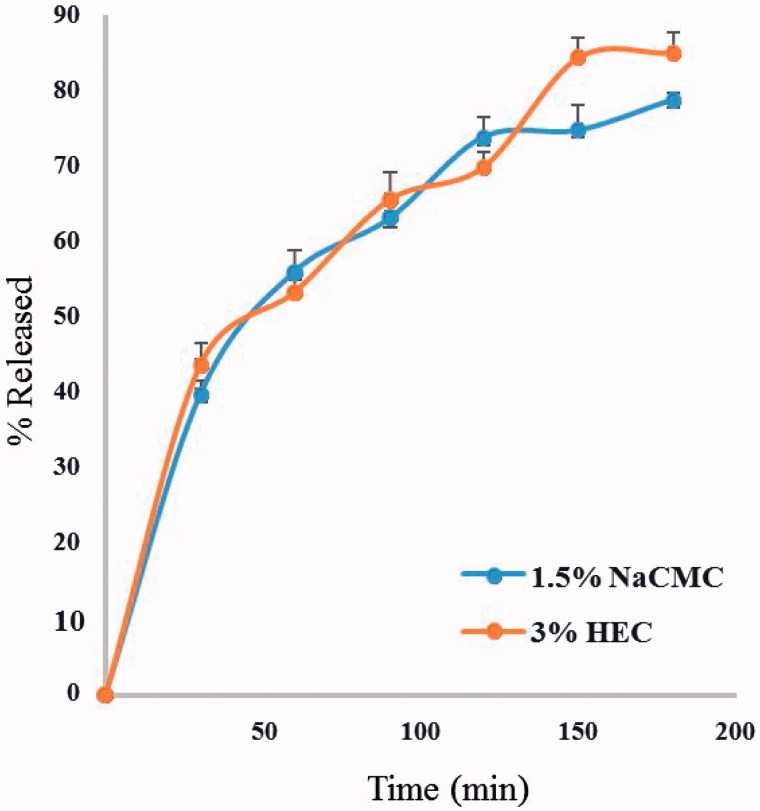
Release of total hypericins from niosomal gels containing 1.5% NaCMC and 3% HEC as polymers.

The niosomal gels were prepared using both 3% HEC and 1.5% NaCMC as polymers, due to the high extent of drug release and the proper physical and rheological characteristics of their hydrogels (results not shown). The *in vitro* drug release (as total hypericins) from both niosomal gels showed similar rates, as well as extent of drug released after 180 min (85.0 ± 4% and 78.8 ± 1% for 3% HEC and 1.5% NaCMC, respectively) (Figure 4). The kinetic analysis of the drug release data revealed that the release of *Hypericum* from all gels followed the Higuchi diffusion mechanism. Niosomal gel prepared using 1.5% NaCMC was chosen as the topical drug delivery system for the *in vivo* trials, based on its drug release data and lower polymer content.

#### *In vivo* wound healing effect

Significant wound closure was recorded on day 14 in the group receiving the niosomal gel 1.5% NaCMC (93.56 ± 5.4%) compared to the control and the Panthenol**®** treated groups (84.32 ± 3.0 and 88.33 ± 1.9%, respectively) (Table 1S).

A significant decrease in the inflammatory cell count was recorded in the group receiving the niosomal gel 1.5% NaCMC (18.4 ± 5.3) at the 7th day post wounding. On the other hand, a significant increase in the inflammatory cell count was recorded in the control and Panthenol**®** treated groups (68.4 ± 2.2 and 61.2 ± 4.2, respectively). Thus the niosomal topical formulation made a marked regression in the inflammatory phase and enhanced the early beginning of the proliferative phase of wound healing, compared to the control and Panthenol-treated groups.

#### Histopathology of the wound

Healing is a systematic process which is explained in terms of four overlapping phases: hemostasis, inflammation, proliferation, and maturation. Platelets play a crucial role in clot formation during hemostasis, while inflammatory cells clear injured tissue during the inflammatory phase. Epithelialization, fibroplasia, and angiogenesis occur during the proliferative phase, while the granulation tissue forms. Collagen forms tight cross-links increasing the tensile strength of the scar during the maturation phase.

**Figure 5.**
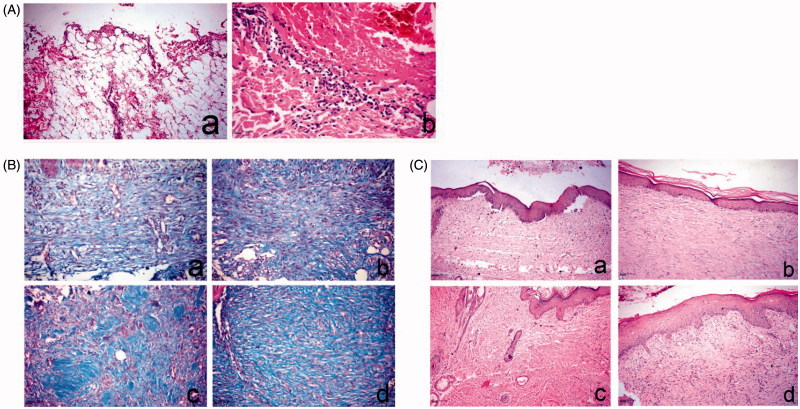
Histopathology of skin wound of dog. (A) At day 0 stained with H&E. a, large wound gap with complete loss of epidermal and dermal cell layer and exposure of the subcutaneous tissue; b, thrombus formation at the margin of the wound intensely infiltrated by polymorph nuclear cells associated with extravagated RBC’s. (B) At day 14, stained with Masson’s trichrome. a, control group showing faint blue stained collagen fibers; b, Panthenol-treated group showing faint blue stained collagen fibers; c and d, *Hypericum* niosomal gel 1.5% NaCMC-treated group showing intense blue well-organized collagen bundles. (C) At day 21, stained with H&E. a, control group showing closure of the wound gap with attenuated epidermal differentiation; b, Panthenol-treated group showing re-epithelization and keratin formation; c and d, *Hypericum* niosomal gel 1.5% NaCMC-treated group showing fully re-epithelialized wound with dermal reconstruction, restoration of skin appendages and appearance of hair follicles (arrow) as well as marked contraction of the wound opening.

In this study, histopathological examination of the skin wound at day 0 showed no significant differences between all groups, as the lesions were characterized by large wound gaps with complete loss of epidermal and dermal cell layers and exposure of the subcutaneous tissue (Figure 5(A)). On the 7th day, the control and Panthenol treated groups revealed extensive edema, hemorrhage, necrosis and massive inflammatory cell infiltration, mostly polymorphnuclear leukocytes. On the other hand, angiogenesis and fibroblastic proliferation were remarkably present in the niosomal gel 1.5% NaCMC treated groups. Masson’s trichrome stain revealed a separation of the fibroblasts by scanty collagen bundles in both control and Panthenol samples. The *Hypericum*-treated group showed a marked increase in collagen density with diffuse accumulation of blue-stained collagen bundles.

On day 14, the Panthenol treated group showed proliferation and migration of the basal keratinocytes at the wound edge associated with extensive edema, as well as fibroblastic proliferation with the formation of collagen fibers. The niosomal gel 1.5% NaCMC treated groups revealed enhanced wound healing denoted by remarkable hyperplastic epidermis at the wound edges and formation of well-organized collagen fibers that appeared blue in Masson’s trichrome stained sections (Figure 5(B)). On the 21st day, the control group showed closure of the wound gap with attenuated epidermal differentiation which was restricted to the basal layer, and skin appendages were not restored. The Panthenol treated group showed more or less the same condition with re-epithelization and keratin formation (Figure 5(C)). The niosomal gel 1.5% NaCMC-treated groups showed complete re-epithelization, formation of new matrix fibers and significant reduction in the wound size, as well as restoration of skin appendages and appearance of hair follicles, marking the termination of the healing process (Figure 5(C)).

## Discussion

*Hypericum perforatum* has been traditionally used for external applications and several studies have reported its use as antimicrobial, antioxidant, anti-inflammatory, anticancer and for wound healing (Wölfle et al., 2014). It is generally prepared in the form of oils (*Oleum Hyperici*) (German Pharmacopoeia 2014) and tinctures. The oil preparations have been extensively studied, but the literature lacks details on the content of active constituents and formulation of the hydroalcoholic preparations (EMEA, 2009). Moreover there is a great variability in the concentrations of these active constituents depending on the geographical origin of the plant, the harvest time and the method of extraction (Isacchi et al., 2007). Therefore, in order to ensure reproducibility of the *in vivo* experiments, detailed description of the extraction method should be provided and a chromatographic finger print of the used extracts must be established. It was previously reported that at least 60% of ethanol/H_2_O is required to obtain both the very lipophilic hyperforin and the hydrophilic polyphenolic compounds (Wölfle et al., 2014). Therefore, 80% ethanolic extract was used in our study after proving that it produced higher yield at 70 °C than at room temperature, and then the 80% methanolic extract (Figure 1 S). Also a quantitative phytochemical analysis of the four extracts revealed that the 80% ethanolic extract contained the highest concentrations of the active constituents (Figure 2(A)). So it was prepared on a pilot scale using DIG-MAZ which provided a well described extraction method ensuring reproducibility of the extract (HE). An HPLC-DAD chromatographic finger print of HE was established (Figure 2(B)) and the extract was standardized to contain specific concentrations of the required active constituents; hyperforin, hypericin and polyphenolic compounds. Several topical formulations of *Hypericum* were developed as gels, ointments, creams and lotions, for inflammation and atopic dermatitis, however they were standardized to either hyperforin and hypericin, or hyperforin alone (Schempp et al., 2000; Schempp et al., 2003). A holistic chromatographic profile of *Hypericum* extract was established for the first time in an *in vivo* study (Figure 2(B)) which ensures reproducibility of the results, efficacy of the extract and better ability to monitor the extract stability. The very lypophilic hyperforin is known for its antimicrobial, anti-inflammatory, antioxidant and keratinocytes stimulation activities. The lypophilic hypericin is known for its antimicrobial activity. The hydrophilic flavonoids and polyphenols are strong antioxidants, and chlorogenic acid enhances epithelialization and cellular proliferation (Cecchini et al., 2007; Koeberle et al., 2011; Rainha et al., 2011; Chen et al., 2013). Therefore the HE extract was standardized to contain 3.4 ± 4, 1.1 ± 3, 0.5 ± 2 and 2.8 ± 2% w/w of rutin, chlorogenic acid, quercitrin and hyperforin, respectively, and 0.51 ± 3% w/w of total hypericins. HE was incorporated for the first time into a niosomal drug delivery system which is capable of entrapping both lypophilic and hydrophilic active constituents (Table 1(B)). Several niosomal systems were prepared where niosome F1 (Span 20: cholesterol, 1:1) showed relatively the highest content of active constituents and 80% of drug entrapment calculated as total hypericins. TEM micrographs of optimum niosomal formula (F1) (Figure 3) confirmed the formation of niosomal vesicles with bilayer membrane. Niosomal gels were prepared using different polymers where 3% HEC and 1.5% NaCMC were chosen due to the high extent of drug release and the proper physical and rheological characters of their hydrogels. The niosomal gel prepared using 1.5% NaCMC was tested in the *in vivo* wound healing experiments on dogs due to its extent of drug release (78.8 ± 1% after 180 min) and lower polymer content. It caused a significant decrease in the inflammatory cell count on day 7 as well as a significant wound closure after 14 days compared to the control and Panthenol**®** treated groups. The niosomal gel 1.5% NaCMC treated groups enhanced wound healing denoted by remarkable hyperplastic epidermis at the wound edges and formation of well-organized collagen fibers on day 14 (Figure 5(B)). It showed complete re-epithelization, significant reduction in the wound size as well as restoration of skin appendages and appearance of hair follicles which was a mark for the termination of the healing process (Figure 5(C)).

## Conclusions

The present study provided a well-described pilot scale extraction method of *H. perforatum* using 80% ethanol at 70 °C. A holistic chromatographic profile for the extract was established and standardized for the first time to its known active constituents, contributing to wound healing (phloroglucinols, naphthodianthrones, flavonoids and polyphenols). The standardized extract was incorporated into a niosomal topical gel which was able to entrap reasonable concentrations of both lypophilic and hydrophilic active constituents. The prepared niosomal gel 1.5% NaCMC showed significantly high effects in the wound treatment of 16 adult mongrel dogs, compared to Panthenol, assuring the synergistic activity of hyperforins, hypericins, flavonoids and polyphenolic compounds. Further studies are needed to test the stability of the niosomal gels.

## Supplementary Material

IDRD_Motaal_et_al_Supplemmetal_Content.docx

## References

[CIT0001] Cecchini C, Cresci A, Coman MM, et al (2007). Antimicrobial activity of seven *Hypericum* entities from central Italy. Planta Med 73:564–6. 17516331 10.1055/s-2007-967198

[CIT0002] Chen WC, Liou SS, Tzeng TF, et al (2013). Effect of topical application of chlorogenic acid on excision wound healing in rats. Planta Med 79:616–21. 23568627 10.1055/s-0032-1328364

[CIT0003] EMEA (European Medicines Agency) (2009). HMPC assessment report on *Hypericum perforatum* L., Herba. EMA/HMPC/101303/2008. London: EMEA.

[CIT0004] Filippini R, Piovan A, Borsarini A, et al (2010). Study of dynamic accumulation of secondary metabolites in three subspecies of *Hypericum perforatum*. Fitoterapia 81:115–9. 19686808 10.1016/j.fitote.2009.08.002

[CIT0005] German Pharmacopoeia (2014). EB6 (Deutsches Arzneibuch. Suppl., 6th edition), (2014). Bonn: Phytopharmaceutical Corporation; 1941: 409.

[CIT0006] Ghaisas MM, Kshirsagar SB, Sahane RS. (2014). Evaluation of wound healing activity of ferulic acid in diabetic rats. Int Wound J 11:523–32. 23236955 10.1111/j.1742-481X.2012.01119.x PMC7950521

[CIT0007] Handa T, Takeuchi H, Ohokubo Y, et al (1987). Lyophilized liposomes prepared by a modified reversed-phase evaporation method. Chem Pharm Bull 35:748–55.

[CIT0008] Isacchi B, Bergonzi MC, Carnevali F, et al (2007). Analysis and stability of the constituents of St. John’s wort oils prepared with different methods. J Pharm Biomed Anal 45:756–61. 17920801 10.1016/j.jpba.2007.08.025

[CIT0009] Koeberle A, Rossi A, Bauer J, et al (2011). Hyperforin, an anti-inflammatory constituent from St. John’s Wort, Inhibits Microsomal Prostaglandin E(2) Synthase-1 and Suppresses Prostaglandin E(2) Formation in vivo. Front Pharmacol 2:7. 21687502 10.3389/fphar.2011.00007 PMC3108608

[CIT0010] Miraldi E, Biagi M, Giachetti D. (2006). Chemical constituents and effects of topical application of *Oleum Hyperici* on skin sensitivity to simulated sun exposure. Nat Prod Commun 1:209–13.

[CIT0011] Moghassemi S, Hadjizadeh A. (2014). Nano-niosomes as nanoscale drug delivery systems: an illustrated review. J Control Release 185:22–36. 24747765 10.1016/j.jconrel.2014.04.015

[CIT0012] Öztürk N, Korkmaz S, Öztürk Y. (2007). Wound-healing activity of St. John’s Wort (*Hypericum perforatum* L.) on chicken embryonic fibroblasts. J Ethnopharmacol 111:33–9. 17156955 10.1016/j.jep.2006.10.029

[CIT0013] Purasa G, Mashala M, Záratea J, et al (2013). A novel cationic niosome formulation for gene delivery to the retina. J Control Release 174:27–36. 24231407 10.1016/j.jconrel.2013.11.004

[CIT0014] Rafiee-Tehrani M, Mehramizi A. (2000). *In vitro* release studies of Piroxicam from oil-in-water creams and hydroalcoholic gel topical formulations. Drug Dev Ind Pharm 26:409–14. 10769782 10.1081/ddc-100101247

[CIT0015] Rainha N, Lima E, Baptista J. (2011). Comparison of the endemic Azorean *Hypericum foliosum* with other Hypericum species: antioxidant activity and phenolic profile. Nat Prod Res 25:123–35. 21246439 10.1080/14786419.2010.512560

[CIT0016] Ruckmani K, Sankar V. (2010). Formulation and optimization of *Zidovudine niosomes*. AAPS PharmSciTech 11:1119–27. 20635228 10.1208/s12249-010-9480-2 PMC2974162

[CIT0017] Schempp CM, Lüdtke R, Winghofer B, et al (2000). Effect of topical application of *Hypericum perforatum* extract (St. John’s wort) on skin sensitivity to solar simulated radiation. Photodermatol Photoimmunol Photomed 16:125–8. 10885442 10.1034/j.1600-0781.2000.d01-18.x

[CIT0018] Schempp CM, Windeck T, Hezel S, et al (2003). Topical treatment of atopic dermatitis with St. John’s wort cream – a randomized, placebo controlled, double blind half-side comparison. Phytomedicine 10:31–7. 12807340 10.1078/1433-187x-00306

[CIT0019] Süntar IP, Akkol EK, Yılmazer D, et al (2010). Investigations on the *in vivo* wound healing potential of *Hypericum perforatum* L. J Ethnopharmacol 127:468–77. 19833187 10.1016/j.jep.2009.10.011

[CIT0020] Tawaha K, Gharaibeh M, El-Elimat T, et al (2010). Determination of hypericin and hyperforin content in selected Jordanian *Hypericum* species. Ind Crop Prod 32:241–5.

[CIT0021] WHO (2002). WHO-monographs on selected medical plants. Geneva: World Health Organization.

[CIT0022] Wölfle U, Seelinger G, Schempp CM. (2014). Topical application of St. John’s wort (*Hypericum perforatum*). Planta Med 80:109–20. 24214835 10.1055/s-0033-1351019

