# Supplementary material for: An in vivo study of Hypericum perforatum in a niosomal topical drug delivery system
Source: Drug Deliv. 2018 Jan 31;25(1):417–25. doi: 10.1080/10717544.2018.1431977 (PMC6058565; doi:10.1080/10717544.2018.1431977)
Supplement: IDRD_Motaal_et_al_Supplemmetal_Content.docx [file IDRD_A_1431977_SM8637.docx]

Supplementary figure and table

Fig 1S. The yield % of different extracts using accelerated solvent extraction ASE^®^100.

MeOH, methanol; rt, room temperature; EtOH, ethanol.

Table 1S. Percentage closure of full thickness wound area (cm^2^) on circular excision wound model in dogs.

| **Day** | | | | | **Group** |
| --- | --- | --- | --- | --- | --- |
| **14** | **9** | **7** | **5** | **3** |  |
| 84.32 ± 3.0 | 81.84 ± 6.0 | 63.62 ± 4.2 | 52.92 ± 2.5 | 36.88 ± 6.8* | **Control** |
| 88.33 ± 1.9 | 81.38 ± 2.2 | 63.43 ± 5.3 | 52.96 ± 0.8 | 42.67 ± 6.2 | **Panthenol®** |
| 93.56 ± 5.4 | 87.57 ± 3.5 | 74.58 ± 6.4 | 56.93 ± 2.3 | 45.19 ± 9.1 | **Hypericum niosomes 1.5% NaCMC gel** |

*Data represented as Mean ± SE (n = 5).
